# Efficacy and Safety of Prophylactic Vaccines against Cervical HPV Infection and Diseases among Women: A Systematic Review & Meta-Analysis

**DOI:** 10.1186/1471-2334-11-13

**Published:** 2011-01-12

**Authors:** Beibei Lu, Ambuj Kumar, Xavier Castellsagué, Anna R Giuliano

**Affiliations:** 1 Risk Assessment, Detection and Intervention Program, H. Lee Moffitt Cancer Center and Research Institute, Tampa, FL, USA; 2 Center for Evidence Based Medicine and Outcomes Research, College of Medicine, University of South Florida, Tampa, FL, USA; 3 Cancer Epidemiology Research Program, Institut Català d'Oncologia (ICO), IDIBELL, CIBER-ESP, L'Hospitalet de Llobregat, Barcelona, Spain

## Abstract

**Background:**

We conducted a systematic review and meta-analysis to assess efficacy and safety of prophylactic HPV vaccines against cervical cancer precursor events in women.

**Methods:**

Randomized-controlled trials of HPV vaccines were identified from MEDLINE, Cochrane Central Register of Controlled Trials, conference abstracts and references of identified studies, and assessed by two independent reviewers. Efficacy data were synthesized using fixed-effect models, and evaluated for heterogeneity using I^2 ^statistic.

**Results:**

Seven unique trials enrolling 44,142 females were included. The fixed-effect Relative Risk (RR) and 95% confidence intervals were 0.04 (0.01-0.11) and 0.10 (0.03-0.38) for HPV-16 and HPV 18-related CIN2+ in the per-protocol populations (PPP). The corresponding RR was 0.47 (0.36-0.61) and 0.16 (0.08-0.34) in the intention-to-treat populations (ITT). Efficacy against CIN1+ was similar in scale in favor of vaccine. Overall vaccines were highly efficacious against 6-month persistent infection with HPV 16 and 18, both in the PPP cohort (RR: 0.06 [0.04-0.09] and 0.05 [0.03-0.09], respectively), and the ITT cohorts (RR: 0.15 [0.10-0.23] and 0.24 [0.14-0.42], respectively). There was limited prophylactic effect against CIN2+ and 6-month persistent infections associated with non-vaccine oncogenic HPV types. The risk of serious adverse events (RR: 1.00, 0.91-1.09) or vaccine-related serious adverse events (RR: 1.82; 0.79-4.20) did not differ significantly between vaccine and control groups. Data on abnormal pregnancy outcomes were underreported.

**Conclusions:**

Prophylactic HPV vaccines are safe, well tolerated, and highly efficacious in preventing persistent infections and cervical diseases associated with vaccine-HPV types among young females. However, long-term efficacy and safety needs to be addressed in future trials.

## Background

Oncogenic HPV infection is the necessary cause of cervical cancer [1]. Worldwide 70% of invasive cervical cancer cases are caused by Human Papillomavirus (HPV) 16 or 18, with HPV 16 being the most common type, detected in 55% of cases, followed by HPV 18, in 15% of cases [2]. Other oncogenic HPV types including 31, 33, 35, 45, 52 and 58 that are phylogenetically related to HPV 16 and 18 account for an additional 18% of all cases. Results from randomized controlled trials (RCTs) of prophylactic HPV vaccines have shown consistently high efficacy in preventing infection and subsequent precancerous cervical lesions associated with vaccine-type oncogenic HPV (HPV 16 and 18) as well as phylogenetically-related oncogenic HPV types. However, due to variability in populations studied, vaccine composition, and efficacy populations defined in different analyses, understanding the published results can prove to be challenging. There is also a need in public at large, including families of targeted women and health care providers, to seek information to support their vaccine decisions.

Two meta-analyses have been published evaluating prophylactic L1 VLP-based HPV vaccine efficacy and safety [3,4]. Few reports have also presented similar data from individual RCTs [5,6]. However, these studies had limitations in addressing multiple clinical endpoints assessed in RCTs [3], and different virus-like-particle (VLP) compositions of individual vaccines [4]. In addition, since the publication of the aforementioned meta-analyses [3,4], newer RCTs with reports on cross-protection have been published. The present study aims to provide a comprehensive assessment of vaccine safety and efficacy against multiple virological and clinical endpoints using the techniques of systematic review and meta-analysis.

## Methods

### Identification and Eligibility of Relevant Studies

A systematic search of MEDLINE, Cochrane Library and the Cochrane Central Register of Controlled Trials was conducted to identify reports of RCTs of prophylactic HPV vaccines published up to July 31^st^, 2009, using a combination of index terms: "Human Papillomavirus", "Papillomavirus vaccines", "Randomized controlled trials", and "Controlled clinical trials". Additional relevant studies were sought through hand search of conference abstract books from IPVS (International Papillomavirus Society) from 2006 to 2009, and bibliographies of obtained studies.

### Inclusion and Exclusion Criteria

RCTs published in English of L1 VLP-based HPV vaccines that were conducted among women and presented measurement of prophylactic efficacy against HPV infection or diseases of interest were included. Trials reporting male vaccination or therapeutic vaccination were excluded. Additionally, we excluded ad hoc subgroup analyses of existing RCTs or combined analyses of multiple RCTs. In the case a RCT reported both interim and final analyses, data from final analyses with complete follow-up were used.

### Outcomes

High-grade cervical lesions or worse (CIN2+), including Cervical Intraepithelial Neoplasia (CIN) grade 2-3, Adenocarcinoma in Situ (AIS), and cervical carcinoma, was the recommended endpoint for establishing efficacy of prophylactic HPV vaccines by WHO and consistently reported in most trials. It was, therefore, chosen as the primary endpoint for efficacy in this review. As persistent HPV infection is an obligate precursor of CIN2-3 and cervical cancer [7], type-specific persistent infections was chosen as the secondary endpoint for efficacy. Furthermore, management of cervical dysplasia of any grade is associated with substantial health care cost in developed countries. Efficacy estimates for CIN 1 or worse (CIN1+) can provide insight for potential impact of vaccination on health care cost. CIN1+ was hence also evaluated as the secondary endpoint. In addition, we examined occurrence of adverse events (AE) and vaccine-related adverse events for assessment of vaccine safety.

### Data Extraction

Data were extracted by two independent reviewers (BL and AK) using a standardized data extraction form. Any discrepancies were resolved by consensus or in consultation with a third reviewer. We extracted detailed information on trial design, inclusion/exclusion criteria, participant characteristics, vaccine and placebo administered, trial endpoints, efficacy populations, and methodological quality from all included trials.

### Statistical Analysis

Effect sizes were summarized as Risk Ratios (RRs) and associated 95 percent confidence intervals. A RR less than one suggested vaccine protection against a specific virological or clinical endpoint. Consistent with definitions used in vaccine RCTs, efficacy was estimated as [1-RR] and expressed as percentage. Three types of populations were defined for efficacy analyses: The intention-to-treat population (ITT), the modified intention-to-treat population (MITT), and the per-protocol population (PPP). Vaccine efficacy was assessed in ITT and PPP cohorts respectively. Efficacy estimates from MITT cohorts were used in the absence of ITT estimates. Vaccine safety was primarily evaluated in ITT cohorts among women who received at least one injection of vaccine or control and had available follow-up, except in one trial [8,9]. A fixed effect model was applied to obtain pooled estimates of vaccine efficacy and safety. Heterogeneity between studies was assessed using the Cochrane's Q and I^2 ^statistics [10]. Sensitivity analyses according to key methodological quality domains and study characteristics were planned *a priori *to explore possible sources of heterogeneity as well as to assess the robustness of the observed results. The possibility of publication bias was assessed using the Begg and Egger funnel plot method [11,12]. The systematic review was performed according to the standards recommended by Cochrane Collaboration [13]. All analyses were performed in RevMan statistical software [14] following the PRISMA guidelines [15].

## Results

### I. Selection of Studies

**Figure 1:**
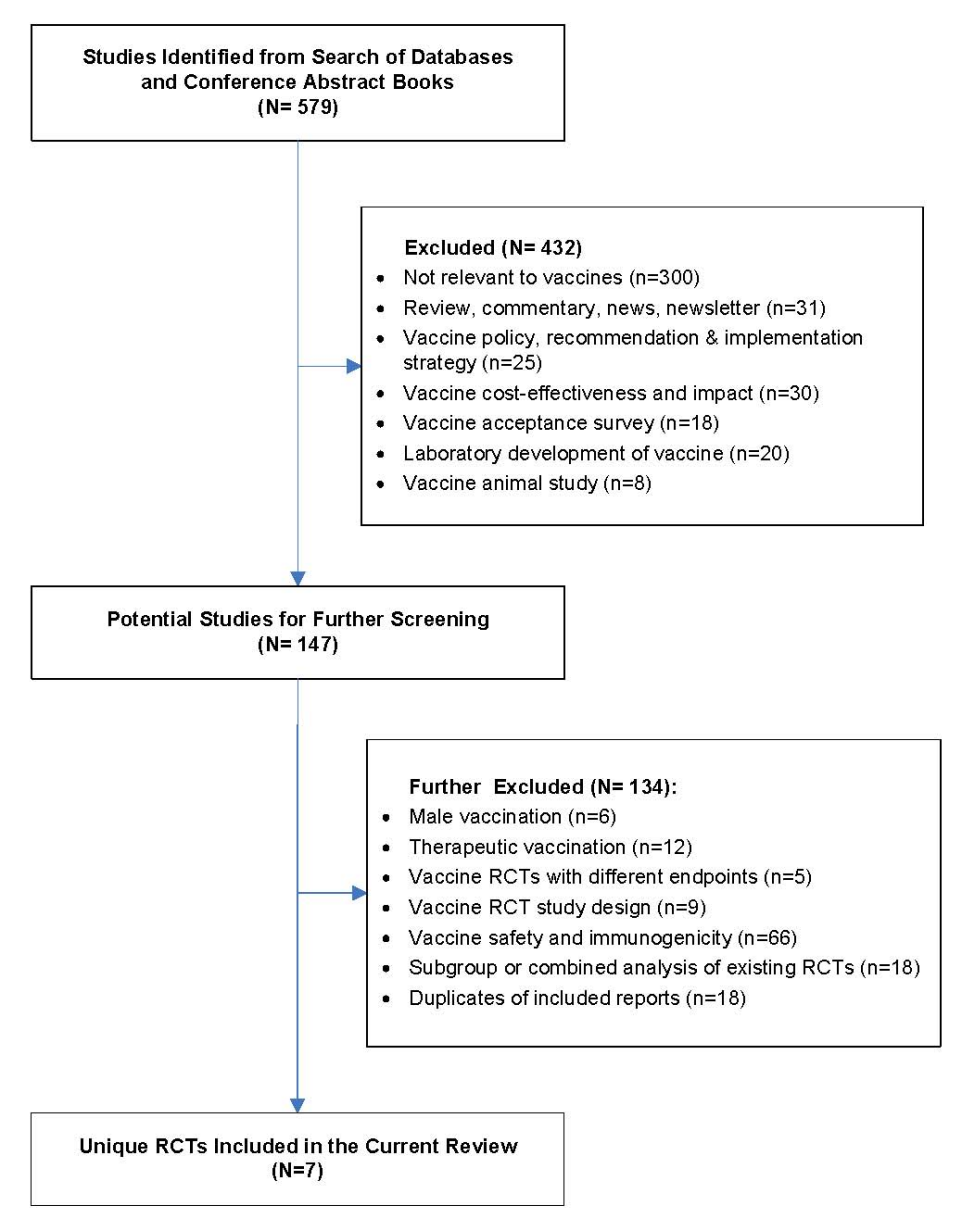
**Inclusion and exclusion of trials in study selection**. RCTs, randomized controlled trials.

Study identification and selection was demonstrated in the flow diagram in Figure 1. Of 579 publications identified through an initial search of databases and conference abstracts, 432 were excluded for reasons elaborated in Figure 1. In total, thirteen publications representing seven unique RCTs met the eligibility criteria and were included in the current review.

### II. Characteristics of Included Trials and Trial Participants

Characteristics of RCTs included in the current review are summarized in Table 1. Unique trials were identified by first authors of associated publication(s). The majority of trials were multinational trials. Eligible participants were non-pregnant women aged 15 to 44 who had 6 or fewer lifetime sexual partners at enrollment and had no history of abnormal Pap smears. Three prophylactic vaccines were evaluated in the seven trials: The bivalent vaccine (containing HPV 16 and 18 VLPs) from GlaxoSmithKline in two trials; the monovalent (containing HPV 16 VLPs) and quadrivalent vaccine (containing HPV 6, 11, 16 and 18 VLPs) from Merck in one and four trials, respectively. Uniform to all trials, vaccines or controls were administered in a three-dose regimen within a 6-month time frame. Proprietary adjuvant was used with each type of vaccine to enhance immunogenicity. All trials used placebo as the comparator except for two in which all or part of the control group received hepatitis A vaccine [16], or placebo plus hepatitis B vaccine [17]. Participants were tested for DNA evidence of HPV infection every 6 months and for cytological abnormality every 6 or 12 months. The length of trials ranged from 26 to 60 months.

**Table 1:** Characteristics of randomized controlled trials included in the review.

	Koutsky & **Mao et al**[28,29]		Harper **et al **[8,9]		Villa **et al **[23,30]		FUTURE I [17,31,32]		FUTURE II [25,31,32]		PATRICIA [16,33]		Muñoz **et al **[18]	
Phase	III		III		II		III		III		III		III	
No. of study sites	16		32		5		62		90		135		38	
Countries included	1		3		5		16		13		14		7	
Year of study enrollment	10/1998-11/1999		11/2003-07/2004		Not reported		01/2002-03/2003		06/2002-05/2003		05/2004-06/2005		06/2004-04/2005	
Funding source	Merck		GlaxoSmithKline		Merck		Merck		Merck		GlaxoSmithKline		Merck	
**Inclusion Criteria**														
Age	16-25		15-25		16-23		16-24		15-26		15-25		24-45	
Lifetime no. of sexual partners	≤ 5		≤ 6		≤ 4		≤ 4		≤ 4		≤ 6		No restriction	
**Exclusion Criteria**	Pregnancy, history of abnormal Pap smear		History of abnormal Pap smear, or ablative or excisional treatment of cervix; ongoing treatment for external condylomata; seropositive for HPV 16 or 18; DNA positive for any of 14 HR HPV in past 90 days		Pregnancy, history of abnormal Pap smear		Pregnancy, history of abnormal Pap smear or genital warts		Pregnancy, history of abnormal Pap smear		History of colposcopy, pregnancy, breastfeeding, autoimmune diseases or immunodeficiency		Pregnancy, history of genital warts, present or past cervical disease, immunocompromised	
**Intervention & Comparator**														
Vaccine component	HPV 16 VLPs		HPV 16, 18 VLPs		HPV 6, 11, 16, 18 VLPs		HPV 6, 11, 16, 18 VLPs		HPV 6, 11, 16, 18 VLPs		HPV 16, 18 VLPs		HPV 6, 11, 16, 18 VLPs	
VLP amount (μg)	40		20/20		20/40/40/20		20/40/40/20		20/40/40/20		20/20		20/40/40/20	
Vaccine adjuvant	225 μg AAHS		AS04 (500 μg/50 μg)		225 μg AAHS		225 μg AAHS		225 μg AAHS		AS04 (500 μg/50 μg)		225 μg AAHS	
Comparator	Placebo		Placebo		Placebo		* Placebo/Placebo+Hepatitis B vaccine		Placebo		Hepatitis A vaccine		Placebo	
Comparator adjuvant	225 μg AAHS		500 μg aluminium hydroxide		225 or 450 μg AAHS		225 μg AAHS		225 μg AAHS		500 μg aluminium hydroxide		225 μg AAHS	
Administration schedule	month 0, 2, 6		month 0, 1, 6		months 0, 2, 6		month 0, 2, 6		month 0, 2, 6		month 0, 1, 6		month 0, 2, 6	
**Clinical Protocol**														
Frequency of HPV DNA test	6 month interval		6 month interval		6 month interval		6 month interval		6 month interval		6 month interval		6 month interval	
Frequency of cytology test	6 month interval		6 month interval		6 month interval		6 month interval		12 month interval		12 month interval		6 month interval	
Length of trial (months)	41.0		Initial trial: 27 Follow-up study: 53		Initial trial: 36 Follow-up study: 60		36.0 (mean)		36.0 (mean)		39.4 (mean)		26.4 (mean)	
**Endpoints**														
Primary	Persistent HPV 16 infection		Incidence infection with HPV 16, and/or 18.		Combined incidence of HPV 6, 11, 16 and/or 18-associated 6-month persistent infection, CIN1-3, AIS, VIN1-3, VaIN1-3, external genital warts and cervical, vulvar or vaginal cancer.		Incidence of HPV 6, 11, 16, and/or 18-associated genital warts, CIN1-3, VIN1-3, VaIN1-3, AIS, and cervical, vulvar or vaginal cancer		HPV 16 and/or 18-associated CIN 2-3, AIS and cervical cancer		HPV 16/18-associated CIN2+		Combined incidence of 6-month persistent infection, CIN1-3, VIN1-3, VaIN1-3, AIS, cervical, vulvar or vaginal cancer, and genital warts associated with HPV 6, 11, 16 or 18, or with HPV 16 or 18 alone.	
Secondary	Transient or persistent HPV 16 infection		Persistent infection with HPV 16, 18 or 16/18; HPV 16/18-associated LSIL, HSIL, CIN1-3 and cancer				Combined incidence of HPV 6, 11, 16 and/or 18-associated CIN1-3, AIS and cancer; Persistent infection, CIN1-3 and AIS associated with HPV 31, 33, 45, 52, 58.		Persistent infection, CIN1-3 and AIS associated with HPV 31, 33, 45, 52, 58		Persistent infection with HPV 16, 18 or other oncogenic types; HPV 16/18-associated CIN1+; immunogenicity and safety		Combined incidence of 6-month persistent infection, CIN1-3, VIN1-3, VaIN1-3, AIS, cervical, vulvar or vaginal cancer, or genital warts associated with HPV 6 or 11	
**Populations for Efficacy Analysis**														
Per-protocol population (PPP)	All subjects that received 3 doses of vaccine/placebo; DNA negative for HPV 16 in cervical swab and biopsy from day 1 to month 7; seronegative for HPV 16 on day 1; had no protocol violation; had a month 7 visit within 14-72 days after the third vaccination		All subjects that received 3 doses of vaccine/placebo; DNA negative for 14 HR HPV on day 1; cytologically negative and seronegative for HPV 16 and 18 on day 1; had no protocol violation		All subjects that received 3 doses of vaccine/placebo within a year; seronegative and DNA negative for HPV 6, 11, 16 or 18 on day 1; remained DNA negative for the same HPV type(s) through month 7; had no protocol violation		All subjects that received 3 doses of vaccine/placebo within a year; seronegative and DNA negative for HPV 6, 11, 16 or 18 on day 1; remained DNA negative for the same HPV type(s) through month 7; had no protocol violation.†		All subjects that received 3 doses of vaccine/placebo within a year; seronegative and DNA negative for HPV 16 or 18 on day 1; remained DNA negative for the same HPV type(s) through month 7; had no protocol violation.†		All subjects that received 3 doses of vaccine/placebo; seronegative to HPV 16 or 18 on day 1; DNA negative to HPV 16 or 18 on day1 and month 6; had normal or low-grade cytology at baseline, had no protocol violation		All subjects that received 3 doses of vaccine/placebo within a year; seronegative and DNA negative in cervicovaginal swab and/or biopsy samples for HPV 6, 11, 16 or 18 on day 1; remained DNA negative to the same HPV type(s) through month 7; had no protocol violation; had one or more follow-up visits after month 7	
Intention-to-treat (ITT)/Modified Intention-to-treat (MITT) population	MITT2: All subjects that received ≥1 dose of vaccine/placebo.		ITT: All subjects that received ≥1 dose of vaccine/placebo; DNA negative for 14 HR HPV on day 1; had data available for outcome measurement.		MITT: All subjects that received ≥1 dose of vaccine/placebo; seronegative and DNA negative to HPV 6, 11, 16 or 18 on day 1.		ITT: All subjects that had undergone randomization, regardless of their baseline HPV status or evidence of HPV-associated anogenital disease.		ITT: All subjects that had undergone randomization, regardless of their baseline HPV status or evidence of cervical neoplasia		ITT: All subjects that received ≥1 dose of vaccine/placebo; DNA negative to HPV 16 or 18 on day 1; had data available for outcome measurement.		ITT: All subjects that received ≥1 dose of vaccine/placebo; had one or more follow-up visits after day1.	
**Methodological Quality**														
Allocation concealment	Adequate		Adequate		Adequate		Adequate		Adequate		Adequate		Adequate	
Blinding	Adequate		Adequate		Adequate		Adequate		Adequate		Adequate		Adequate	
Dropout/loss-to-follow-up reported	Yes		Yes		Yes		Yes		Yes		Yes		Yes	
Expected efficacy (1-RR)	0.75		0.70		0.80		0.80		0.80-0.90		0.85		0.80	
Sample size calculation performed	Yes		Yes		Yes		Yes		Yes		Yes		Yes	
	α = 0.05 (one-sided)	β = 0.10	α = 0.05 (two-sided)	β = 0.20	α = 0.05 (two-sided)	β = 0.10	α = 0.0125 (one-sided)	β = 0.09	α = 0.02055 (one-sided)	β = 0.10	α = 0.05 (two-sided)	β = 0.06	--	β = 0.13

HR HPV: High-risk HPV includes HPV 16, 18, 31, 33, 35, 39, 45, 51, 52, 56, 58, 59, 66, and 68; CIN: Cervical intraepithelial neoplasia; AIS: Adenocarcinoma in situ; CIN1+: Cervical intraepithelial neoplasia grade 1 or worse, including CIN1-3, AIS and cervical cancer. CIN2+: Cervical intraepithelial neoplasia grade 2 or worse, including CIN2-3, AIS and cervical cancer; LSIL: Low-grade intraepithelial lesion; HSIL: High-grade intraepithelial lesion; VIN: Vulvar intraepithelial neoplasia; VaIN: Vaginal intraepithelial neoplasia. VLPs: Virus-like particles; AAHS: Amorphous aluminium hydroxyphosphate sulphate. AS04: 500 μg aluminum hydroxide and 50 μg 3-O-desacyl-4'-monophosphoryl lipid A; RR: Risk ratio, the ratio of event rates of vaccine and control group.

* A subset of 466 subjects in the treatment arm received quadrivalent vaccine and Hepatitis B vaccine, and 467 subjects in control arm received placebo and Hepatitis B vaccine.

† Per-protocol population for evaluation of cross-protection included subjects who received ≥1 vaccination and, at enrollment were seronegative and DNA negative for each of vaccine HPV types (6, 11, 16, and 18); were DNA negative for each of 10 non-vaccine types (31, 33, 35, 39, 45, 51, 52, 56, 58, and 59); and had a normal Pap test result.

The mean age of participants for the majority of trials was approximately 20 years except in the trial of Munoz and associates where older females were recruited (mean age 34) (Table 2) [18]. The majority of participants in individual trials had two or fewer lifetime sexual partners and used hormonal contraceptives. Approximately 90% of participants in each trial had normal cytology at enrollment.

**Table 2:** Baseline characteristics of participants in the intention-to-treat cohort of randomized controlled trials included in the review.

Characteristics, n (%)	Koutsky & Mao **et al**. §		Harper **et al**.		Villa **et al**.		FUTURE I		FUTURE II		PATRICIA		Muñoz **et al**.	
	
	Vaccine (n = 768)	Control (n = 765)	Vaccine (n = 560)	Control (n = 553)	Vaccine (n = 277)	Control (n = 275)	Vaccine (n = 2723)	Control (n = 2732)	Vaccine (n = 6087)	Control (n = 6080)	Vaccine (n = 9319)	Control (n = 9325)	Vaccine (n = 1911)	Control (n = 1908)
Age, mean (SD)	20.0 (1.6)	20.1 (1.6)	20.4 (2.8)	20.5 (2.7)	20.2 (1.7)	20.0 (1.7)	20.2 (1.8)	20.3 (1.8)	20.0 (2.2)	19.9 (2.1)	20.0 (3.1)	20 (3.1)	34.3 (6.3)	34.3 (6.3)
HPV 16 Positivity														
DNA	--	--	--	--	49 (17.7)^†^	51(18.5)^†^	238 (8.9)	227 (8.4)	543 (9.1)	545 (9.1)	516 (5.6)	478 (5.2)	--	--
Serology	143 (12.0)	166 (13.9)	--	--			312 (11.5)	319 (11.7)	652 (10.7)	688 (11.3)	1544 (16.7)	1552 (16.8)	--	--
HPV 18 Positivity														
DNA	--	--	--	--	17 (6.1)^†^	21 (7.6)^†^	86 (3.2)	83 (3.1)	230 (3.8)	242 (4.0)	215 (2.3)	216 (2.3)	--	--
Serology	--	--	--	--			93 (3.4)	90 (3.3)	227 (3.7)	236 (3.9)	1076 (11.7)	1070 (11.6)		
Lifetime no. of sexual partners	≤ 5		≤ 6		≤ 4		≤ 4		≤ 4		≤ 6		No restriction	
None	38 (5)	34 (4)	90 (16)	85 (16)	17 (6)	16 (6)	--	--	--	--	294 (3)^††^	292 (3)^††^	0 (0)	2 (0.1)
One	218 (28)	200 (26)	197 (35)	188 (35)	80 (29)	88 (32)	--	--	--	--	5862 (63)^††^	5869 (63)^††^	719 (38)	751 (39)
Two/Two to five	173 (23)	173 (23)	259 (46)	242 (45)	73 (26)	75 (27)	--	--	--	--	1114 (12)^††^	1161 (13)^††^	385 (20)	362 (19)
Three/Three or more	138 (18)	131 (17)	--	--	67 (24)	50 (18)	--	--	--	--	636 (7)^††^	595 (6)^††^	229 (12)	223 (12)
Four/Four or more	105 (14)	144 (19)	--	--	40 (14)	46 (17)	--	--	--	--	--	--	142 (7)	130 (7)
Five/Five or more	96 (13)	83 (11)	14 (3)	18 (3)			--	--	--	--	--	--	433 (23)	437 (23)
Smoking status														
Never smoker	--	--	102 (18)	85 (16)	--	--	--	--	--	--	6401 (69)*	6388 (69)*	923 (48)	935 (49)
Former smoker	--	--	164 (30)	138 (26)	--	--	--	--	--	--	2706 (29)*	2726 (29)*	159 (8)	148 (8)
Current smoker	183 (24)	190 (25)	294 (52)	310 (58)	--	--	696 (26)	716 (26)	--	--	--	--	339 (18)	332 (17)
Chlamydia trachomatis														
Negative	--	--	--	--	--	--	2565 (94)	2545 (93)	5723 (94)	5737 (94)	8155 (88)	8188 (88)	--	--
Positive	24 (3)	19 (3)	--	--	--	--	118 (4)	135 (5)	258 (4)	224 (4)	478 (5)	475 (5)	--	--
Contraceptive use														
Barrier	--	--	--	--	63 (23)	76 (28)	872 (32)	874 (32)	--	--	--	--	441 (23)	425 (22)
Hormonal	--	--	--	--	161 (58)	157 (57)	1568 (58)	1539 (57)	3613 (60)	3614 (60)	5544 (59)	5662 (61)	596 (31)	591 (31)
Behavioral	--	--	--	--	48 (17)	48 (17)	487 (18)	498 (18)	--	--	--	--	165 (9)	184 (10)
Other	--	--	--	--	21 (8)	17 (6)	125 (5)	138 (5)	--	--	--	--	748 (39)	749 (39)
Cytological status at entry														
Normal	656 (88)	655 (87)	--	--	--	--	2360 (89)	2326 (88)	5222 (87)	5242 (89)	8395 (90)	8450 (91)	--	--
Abnormal	84 (11)	96 (13)	--	--	--	--	288 (11)	316 (12)	697 (12)	654 (11)	908 (10)	860 (10)	--	--

† Seropositive or DNA positive to HPV 16 or 18 on day 1. †† Reported as "number of sexual partners in past year" in PATRICIA study. § Participant characteristics were presented for the per-protocol population only.

* Never smoker category includes participants who never smoke or smoked for ≤6 months (current/past); Former smoker category includes participants who smoked ≥6 months (current/past) in PATRICIA trial.

Methodological quality was generally high for included trials. All trials demonstrated adequate reporting for allocation concealment, blinding, and drop-outs or loss-to-follow-up. Expected efficacy and sample size estimates were presented for each trial with considerations for dropout or loss-to-follow-up.

The Begg and Egger funnel plot (Additional file 1) for the primary endpoint, CIN2+ associated with HPV 16/18 in the ITT cohorts (*p *= 0.602), indicated no significant publication bias.

### III. Outcomes

#### 1) CIN2+ associated with HPV 16 (Figure 2A)

**Figure 2:**
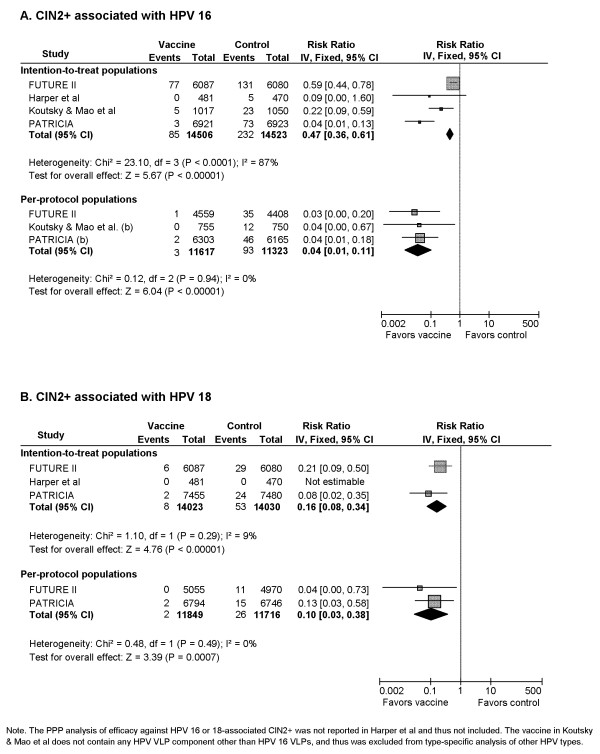
Vaccine efficacy against cervical intraepithelial neoplasia grade 2 or worse (CIN2+) associated with HPV 16 and 18

The pooled RR for 4 RCTs enrolling 28,639 participants was 0.47 (95% CI: 0.36-0.61) in ITT cohorts, corresponding to a pooled efficacy of 53% and indicating a statistically significant benefit with vaccine use. However, there was significant heterogeneity among pooled studies (Cochrane's Q, p < 0.001; I^2 ^= 87%).

Comparable efficacy estimates were reported by PPP cohort of individual trials. The pooled RR for PPP cohorts (3 RCTs, 22,940 participants) was 0.04 (95% CI: 0.01-0.11), showing a statistically significant reduction of 96% in CIN2+ incidence due to vaccination.

#### 2) CIN2+ associated with HPV 18 (Figure 2B)

Analysis using ITT cohorts showed a statistically significant benefit of vaccination with a pooled RR (3 RCTs, 28,053 participants) of 0.16 (95% CI: 0.08-0.34), translating to an 84% protection against HPV 18 associated CIN2+ for vaccine recipients.

Two of the three trials included in the ITT analyses provided extractable data for the PPP analyses. The overall RR for PPP cohorts (2 RCTs, 23,565 participants) was 0.10 (95% CI: 0.03-0.38), equivalent to a 90% protection against HPV 18 associated CIN2+ for vaccine recipients compared with control recipients.

#### 3) CIN1+ associated with HPV 16 (Figure 3A)

**Figure 3:**
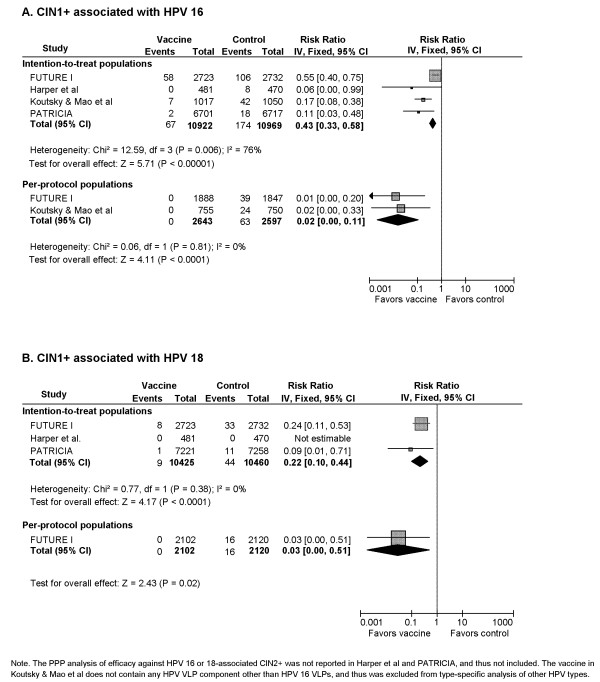
**Vaccine efficacy against cervical intraepithelial neoplasia grade 1 or worse (CIN1+) associated with HPV 16 and 18**.

Vaccines of different compositions demonstrated a statistically significant level of protection against HPV 16-associated CIN 1+ in ITT cohorts. The pooled RR (4 RCTs, 21,891 participants) was 0.43 (95% CI: 0.33-0.58) corresponding to a 57% reduction in CIN1+ incidence for vaccine recipients compared with control recipients. However, there was statistically significant heterogeneity among included trials (Cochrane's Q, p = 0.006; I^2 ^= 76%). The summary RR for PPP cohorts (2 RCTs, 5,240 participants) was 0.02 (95% CI: 0.00-0.11), indicating a 95% reduction in CIN1+ incidence due to vaccine use.

#### 4) CIN1+ associated with HPV 18 (Figure 3B)

HPV vaccine provided a statistically significant protection against HPV 18-associated CIN1+ for vaccine recipients in ITT cohorts with a pooled RR (3 RCTs, 20,885 participants) of 0.22 (95% CI: 0.10-0.44), translating to an efficacy of 78%. Only one of three trials provided efficacy data for PPP cohort, reporting a RR (1 RCT, 4,222 participants) of 0.03 (95% CI: 0.00-0.51), a 97% reduction.

#### 5) Persistent HPV 16 infection of ≥6 months (Figure 4A)

**Figure 4:**
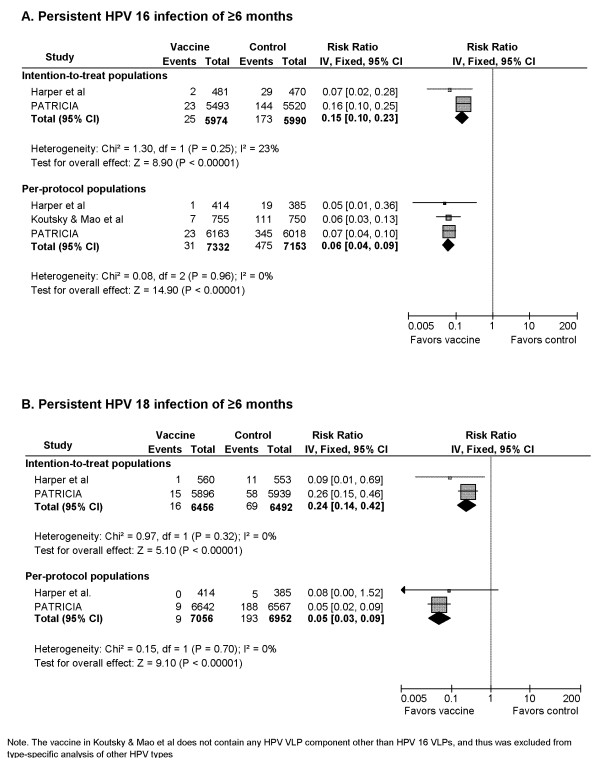
Vaccine efficacy against persistent infection of ≥ 6-months with HPV 16 and 18

Persistent infection of ≥6 months was defined as the detection of same HPV DNA at two or more consecutive visits over a minimum of 4 months from the beginning of case counting. While three RCTs reported efficacy information for PPP cohorts, only two provided information for ITT cohorts. The pooled RR (2 RCTs, 11,964 participants) for the risk of 6-month persistent HPV 16 infection in ITT cohorts was 0.15 (95% CI: 0.10-0.23), equivalent to an 85% of risk reduction for vaccine recipients compared with control recipients.

A statistically significant protection derived from vaccine use was observed in PPP cohorts. The pooled RR (3 RCTs, 14,485 participants) was 0.06 (95% CI: 0.04-0.09), corresponding to a 94% lower risk of persistent HPV 16 infection for vaccine recipients in PPP cohorts.

#### 6) Persistent HPV 18 infection of ≥6 months (Figure 4B)

Vaccines conferred a statistically significant protection against 6-month persistent HPV 18 infection, with a pooled RR of 0.24 (95% CI: 0.14-0.42) for ITT cohorts (2 RCTs, 12,948 participants) and a RR of 0.05 (95% CI: 0.03-0.09) for PPP cohorts (2 RCTs, 14,008), equivalent to a 76% and 95% protection derived from vaccination, respectively.

#### 7) CIN2+ and Persistent Infection of ≥6 Months Associated with HPV 31/33/45/52/58 (Figure 5, 6)

**Figure 5:**
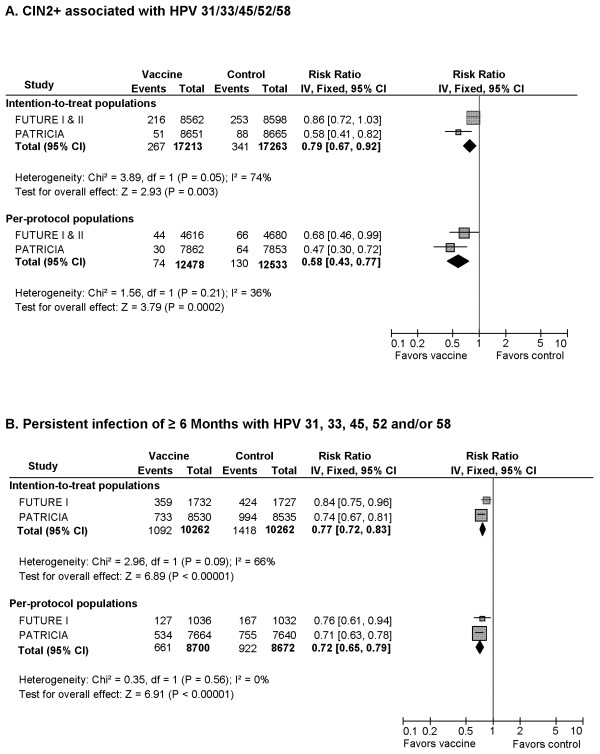
Cross-protection against infections and diseases associated with non-vaccine oncogenic HPV types

**Figure 6:**
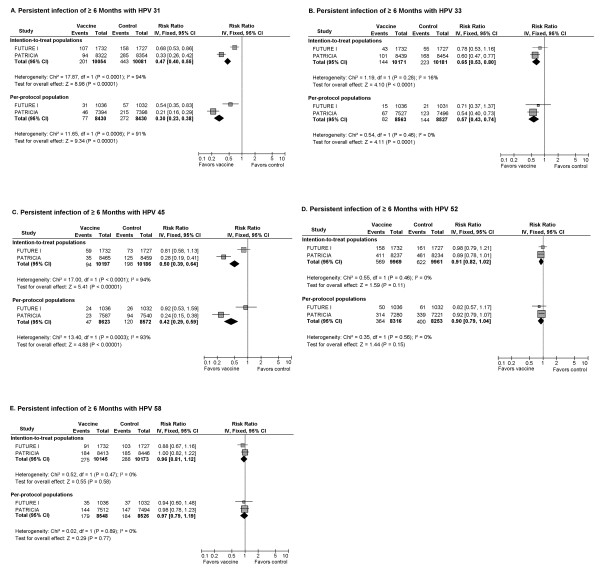
**Cross-protection against persistent infection of **≥ **6-months with HPV 31, 33, 45, 52 and 58**

In ITT analyses, vaccines demonstrated statistically significant reduction (p = 0.003) in the risk of CIN2+ associated with non-vaccine oncogenic HPV types with a pooled RR (3 RCTs, 34,476 participants) of 0.79 (95% CI: 0.67-0.92) (Figure 5A). However, there was borderline significant heterogeneity among included trials (Cochrane's Q, p = 0.05; I^2 ^= 74%). The overall RR was 0.58 (95% CI: 0.43-0.77) for PPP cohorts (3 RCTs, 25,011 participants), indicating a ~40% cross-protection against non-vaccine oncogenic HPV-associated CIN2+.

Two RCTs demonstrated comparable level of protection against 6-month persistent HPV 31/33/45/52/58 infection in ITT and PPP cohorts, respectively (Figure 5B). The pooled RR was 0.77 (95% CI: 0.72-0.85) for ITT cohorts (20,524 participants) and 0.72 (95% CI: 0.65-0.79) for PPP cohorts (17,372 participants) indicating a limited but statistically significant cross-protection.

When cross-protection against 6-month persistent infection was examined by HPV type (Figure 6A-E), prophylactic vaccines were most efficacious in preventing persistent HPV 31 infection (RR: ITT 0.47 and PPP 0.30), followed by persistent infection with HPV 45 (RR: ITT 0.50 and PPP 0.42) and HPV 33 (RR: ITT 0.65 and PPP 0.57). No statistically significant cross-protection against persistent infection with HPV 52 or 58 was detected in individual trials or overall. Significant heterogeneity between included trials was observed in both ITT and PPP analyses of vaccine efficacy against persistent HPV 45 infections (ITT: Cochrane's Q, p < 0.0001, I^2 ^= 94%; PPP: Cochrane's Q, p < 0.0003, I^2 ^= 93%).

### IV. Assessment of Adverse Events (Figure 7)

**Figure 7:**
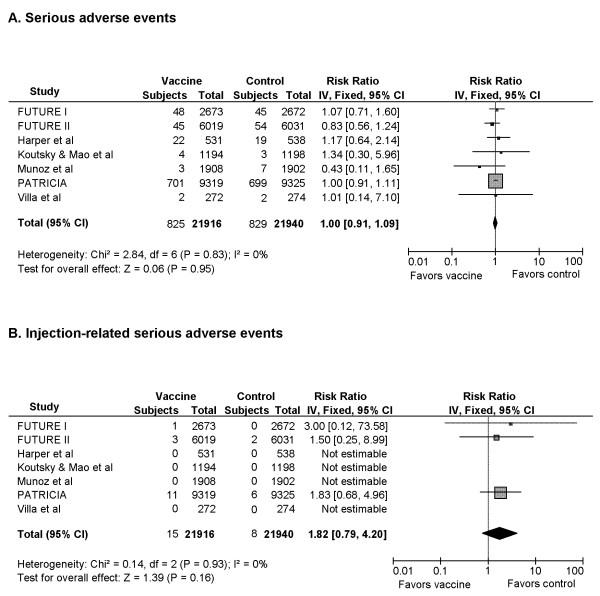
Assessment of Adverse Events

Adverse events (AEs) were monitored by the use of daily vaccination report cards within 15 or 30 days of injection, as well as solicitation throughout the study. Occurrence of AEs was reported in all RCTs. Pain at injection site was the most frequently reported AE ranging from 83.0 - 93.4% in vaccine groups and 75.4 - 87.2% in control groups. Headache and fatigue were the most common vaccine-related systemic AEs observed in approximately 50-60% of all participants. Serious AE reported included abnormal pregnancy outcomes, blood and lymphatic system disorder, hepatobiliary disorder, immune system disorder, cardiac and vascular disorder, gastrointestinal disorder, musculoskeletal and connective tissue disorder, nervous system disorder, psychiatric disorder, renal and urinary disorder, reproductive system and breast disorder, respiratory, thoracic and mediastinal disorder, skin and subcutaneous tissue disorder, neoplasm, infection and infestation, injury, poisoning and procedural complications. The most commonly reported serious AEs were abnormal pregnancy outcomes such as abnormal infant and spontaneous abortion. The pooled RR for participants experiencing one or more serious AEs was 1.00 (95% CI: 0.91-1.09) suggesting a statistically insignificant difference in the risk of serious AEs between vaccine and control groups (Figure 7A).

Serious AEs that were judged to be related to injection included bronchospasm, gastroenteritis, headache, hypertension, injection-site pain, decrease in joint movement at injection site, hypersensitivity to injection, chills, headache and fever. Four of the seven trials reported zero injection-related serious AEs (Figure 7B). Among those reporting vaccine-related serious AEs, the event rate ranged from 0-0.1%. Overall there was no statistically significant difference in the risk for vaccine-related serious AEs between vaccine and control groups (RR, 1.82; 95% CI: 0.79-4.20).

### V. Sensitivity Analyses (Additional file 2)

Sensitivity analysis was performed to identify potential sources of heterogeneity that was observed in the ITT analyses of CIN1+ and CIN2+ associated HPV 16. The included trials did not differ by methodological qualities in terms of allocation concealment, blinding, effect size, power calculation, and participant withdraw and drop-out. Therefore, heterogeneity was unlikely attributed to the methodological quality. We further examined the heterogeneity among included trials according to study characteristics including study population, inclusion/exclusion criteria, intervention, comparator, endpoints chosen and efficacy populations defined, as well as participant baseline characteristics such as age, HPV prevalence and lifetime number of sex partners. The pooled efficacy estimates for FUTURE trials were significantly lower than those for all other RCTs (CIN2+: FUTURE II 0.59, 95% CI: 0.44-0.78 vs. Other RCTs 0.11, 95% CI: 0.05-0.23; CIN1+: FUTURE I 0.55, 95% CI: 0.40-0.75 vs. Other RCTs 0.15, 95% CI: 0.07-0.29). With the FUTURE trials excluded, the observed heterogeneity dissipated for either endpoint (CIN2+: I^2 ^= 59%, p = 0.09; CIN1+; I^2 ^= 0%, p = 0.70). The previously observed heterogeneity and lower efficacy reported by the FUTURE trials was likely due to inclusion of a larger proportion of trial participants already infected with vaccine HPV types at baseline in the ITT cohorts, some of whom may have progressed to cervical neoplasia during the follow-up period.

## Discussion

In summary, the totality of evidence derived from this meta-analysis demonstrated that prophylactic vaccines are highly efficacious in preventing vaccine type HPV infections and associated precancerous cervical lesions. Efficacy against persistent HPV 16 and 18 infections was most impressive, offering 95% efficacy for PPP and 75-85% for ITT cohorts. Vaccines were equally efficacious in preventing HPV 16 and 18 associated CIN1+ with an efficacy of 57-78% for ITT and 97-98% for PPP cohorts. Efficacy was also pronounced for CIN2+ associated with HPV 16 and 18 with over 90% efficacy for PPP and 50% for ITT cohorts.

Overall, a notable higher efficacy was observed in PPP cohorts than in the ITT or MITT cohorts. The discrepancies are likely attributable to differences in the populations evaluated and case counting methods adopted for different types of efficacy analyses. The ITT cohort typically included women who had received ≥1 dose of vaccine or placebo, and had available follow-up data regardless of HPV status at enrollment. A MITT cohort required participants to be virologically and serologically naïve to vaccine HPV types at enrollment, in addition to the requirements set for ITT. In some cases, a normal cytology at enrollment and DNA negativity to other oncogenic HPV types at enrollment were also required. The PPP cohort was, in comparison, more restricted and only included women who received all three doses of vaccine or placebo, were naïve (DNA- and sero-negative) to vaccine HPV types at enrollment and remained DNA-negative through the regimen with no protocol violation. In addition, case counting for ITT or MITT analyses usually started on day 1, instead of one month post dose three in most PPP analyses. The excessive cases observed in ITT cohorts compared with those in PPP cohorts were likely the results of incomplete vaccine regimen and the inclusion of prevalent cases of HPV infection and low-grade cervical diseases at enrollment.

The ITT cohort in the vaccine trials mimics young women in the general population who may have been exposed to vaccine type HPV infection and have less than perfect compliance with vaccination protocol, whereas PPP cohorts approximates pre-sexually active young adolescents naïve to vaccine type HPV with perfect or nearly perfect compliance. The young adolescent group becomes susceptible soon after their initiation of sexual activities, even if they are not engaged in penetrative sexual intercourse [19,20]. The notable difference in prophylactic efficacy against infection and cervical diseases between the ITT and PPP cohorts of young females and the lack of therapeutic efficacy [21] highlight the public health importance of early vaccination of adolescent girls prior to their sexual debut and vaccine compliance.

While sexually naïve young women may derive greater public health benefit from HPV vaccination, vaccine appears to be of benefit to a broad age range of women many of whom may have acquired transient infections in the past or had active infection at the time of vaccination. Preliminary data from the quadrivalent vaccine trial of Muñoz and associates [18] enrolling 3819 24-45 year old women showed a 90% efficacy against combined incidence of vaccine HPV-related 6-month persistent infection, CIN 1-3 or external genital warts in PPP cohorts and a 31% efficacy in the ITT cohort. Recent studies further demonstrated that even among women who had detectable serological evidence of vaccine-HPV infection in the past and no DNA evidence of active infection at enrollment, prophylactic vaccination provides nearly 100 percent protection (95% CI: <0-100) against CIN2+ associated with a vaccine HPV type with which the women had been previously infected [22]. These data suggest that older reproductive-age women can still benefit from prophylactic vaccination.

Whether prophylactic vaccines offer long-term protection remains an important unanswered question. Two included trials offered an extended follow-up of 53 months and 60 months [9,23] and both reported high sustained efficacy against vaccine HPV infection and associated cervical diseases for the extended follow-up phase. The longest follow-up available to date is 8.5 years for the monovalent vaccine trial [24]. The trial reports an efficacy against HPV 16-related CIN2+ of 64% (95% CI: -51-94) for ITT and 100% (95% CI: 29-100) for PPP in the extended follow-up phase among a subset (n = 280) of participants enrolled for the initial trial, comparable with the efficacy of 78% (95% CI: 41-93) for ITT and 100% (95% CI: 65-100) for PPP reported in the initial trial. Future efficacy data from longer-term follow-up of bivalent and quadrivalent vaccine trials are critical to fully address long-term efficacy.

Our results demonstrated statistically significant but limited protection against CIN2+ associated with non-vaccine-type oncogenic HPV that are phylogenetically related to HPV 16 and 18 in both ITT and PPP cohorts (RR: 0.79 in ITT and 0.58 in PPP). Limited cross-protection against persistent infection of ≥6 months was consistently observed for HPV 31, and to a lesser extent against persistent HPV 45 and HPV 33 infection. No statistically significant protection was detected against persistent infection with HPV 52 and 58. The added benefit of cross-protection may result in further reductions in incidence of cervical cancer and precancerous lesions following vaccination. However, whether cross-protection remains present in long-term follow-up, and if present, how the efficacy trend for non-vaccine types compares with that of vaccine HPV types has yet to be determined.

Results of the safety assessment indicated that injection-related local and systemic symptoms were generally mild. The most commonly reported serious AEs were abnormal pregnancy outcomes. Serious AEs that were considered to be vaccine-related were rare. It was noted that data on abnormal pregnancy outcomes were underreported, only available in three of the seven trials included [16,17,25]. The currently licensed prophylactic vaccines evaluated in the review were not recommended by the Advisory Committee on Immunization Practices (ACIP) for pregnant women, although it was suggested that abnormal pregnancy outcomes did not differ significantly between vaccine and control group, and were comparable with those reported in surveillance registries and the literature in a recent publication that combined pregnancy and infant outcomes from five Phase III RCTs [26]. However, pregnancy testing was not required before vaccination by ACIP. Given the high frequency of pregnancy among 15-44 year-old women in the general population (103.2 per 1,000 women) [27] as well as in vaccine trial participants (~20%) [16,26], further data on pregnancy outcomes, and long-term follow-up of live births conceived during trial regimen are needed for a full assessment of vaccine safety and to facilitate women with an informed vaccine decision.

There are several limitations to the evidence presented here. We were not able to evaluate prophylactic efficacy against anogenital warts, vulvar or vaginal diseases associated with vaccine HPV due to the common use of composite endpoints in individual RCTs that combined infections, lesions and cancers of various anogenital sites, and were often incomparable across RCTs [17,18,23]. In addition, we did not evaluate HPV 6 or 11-specific efficacy given that VLP components of HPV 6 and 11 were only included in the quadrivalent vaccine from Merck. Furthermore, we recognize that the pooled efficacy results presented here were derived from susceptible young females with limited sexual exposure to HPV, therefore, they may not be applicable to more mature, sexually active female populations. Lastly, due to the limited number of published trials available to date, we were not able to assess efficacy pertaining to each type of vaccine.

## Conclusion

In conclusion, our review demonstrated that VLP-based prophylactic HPV vaccines are highly efficacious in preventing persistent infection and cervical diseases associated with vaccine HPV types among young female adults. The vaccines were safe and generally well tolerated. Vaccination of adolescent girls prior to sexual debut appeared to be the most effective public health measure for prevention of cervical diseases and cancer. Questions related to long-term efficacy and safety have yet to be addressed.

## Competing interests declaration

Authors declare that (1) No author have received support from any company for the submitted work; (2) XC serves as a consultant to Sanofi Pasteur MSD Ltd. and GlaxoSmithKline, and ARG serves an advisory board member to Merck & Co. Inc.; (3) their spouses, partners, or children have no financial interests that may be relevant to the submitted work; and (4) all authors have no non-financial interests that may be relevant to the submitted work.

## Authors' contributions

BL was responsible for conception and design of the study, conduct of analysis, interpretation of data, and drafting and revision of the manuscript; AK was involved in design of the study, conduct of analysis, interpretation of data and critical revision of the manuscript; XC and ARG participated in design of the study, interpretation of data, and critical revision of the manuscript. All authors have read and approved the final manuscript. Requirement on Ethics Approval: As this analysis is a secondary analysis of published data and uses no identifiable patient information, ethical approval is not required. Data Access: All authors of this manuscript had full access to all of the data (including statistical reports and tables) in the study and can take responsibility for the integrity of the data and the accuracy of the data analysis.

## Pre-publication history

The pre-publication history for this paper can be accessed here:

http://www.biomedcentral.com/1471-2334/11/13/prepub

## Supplementary Material

Additional file 1 **Figure **1**. Assessment of Publication Bias for the Primary Endpoint, CIN2+ Associated with HPV 16 and 18**. Funnel plot for assessment of publication bias. Click here for file

Additional file 2 **Figure **2**. Sensitivity Analysis for Select Endpoints with Significant Heterogeneity**. Forest plots showing results of subgroup analysis for select endpoints. Click here for file
