# Supplementary figures and images for: Efficacy and Safety of Prophylactic Vaccines against Cervical HPV Infection and Diseases among Women: A Systematic Review & Meta-Analysis
Source: BMC Infect Dis. 2011 Jan 12;11:13. doi: 10.1186/1471-2334-11-13 (PMC3034689; doi:10.1186/1471-2334-11-13)

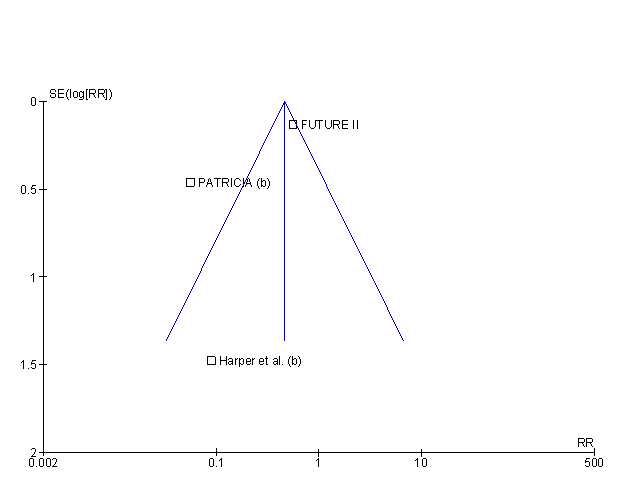

Supplement: Additional file 1 — Figure 1. Assessment of Publication Bias for the Primary Endpoint, CIN2+ Associated with HPV 16 and 18. Funnel plot for assessment of publication bias. [file 1471-2334-11-13-S1.TIFF]
